# Supplementary material for: Nanoengineering of cathode layers for solid oxide fuel cells to achieve superior power densities
Source: Nat Commun. 2021 Jun 25;12:3979. doi: 10.1038/s41467-021-24255-w (PMC8233395; doi:10.1038/s41467-021-24255-w)
Supplement: Supplementary file 1 — Supplementary Information [file 41467_2021_24255_MOESM1_ESM.pdf]

Supplementary Information

Nanoengineering of cathode layers for solid oxide fuel cells to achieve superior power densities

Katherine Develos-Bagarinao[1,2*], *Tomohiro Ishiyama*[2], *Haruo Kishimoto*[1,2], *Hiroyuki Shimada*[3] *and Katsuhiko Yamaji*[2]

[1]Global Zero Emission Research Center, National Institute of Advanced Industrial Science and Technology (AIST), 16-1 Onogawa, Tsukuba, Ibaraki 305-8569, Japan

[2]Research Institute for Energy Conservation, National Institute of Advanced Industrial Science and Technology (AIST), 1-1-1 Higashi, Tsukuba, Ibaraki 305-8565, Japan

[3]Innovative Functional Materials Research Institute, National Institute of Advanced Industrial Science and Technology (AIST), 2266-98 Anagahora, Shimo-shidami, Moriyama-ku, Nagoya, Aichi 463-8560, Japan

*email: develos-bagarinao@aist.go.jp

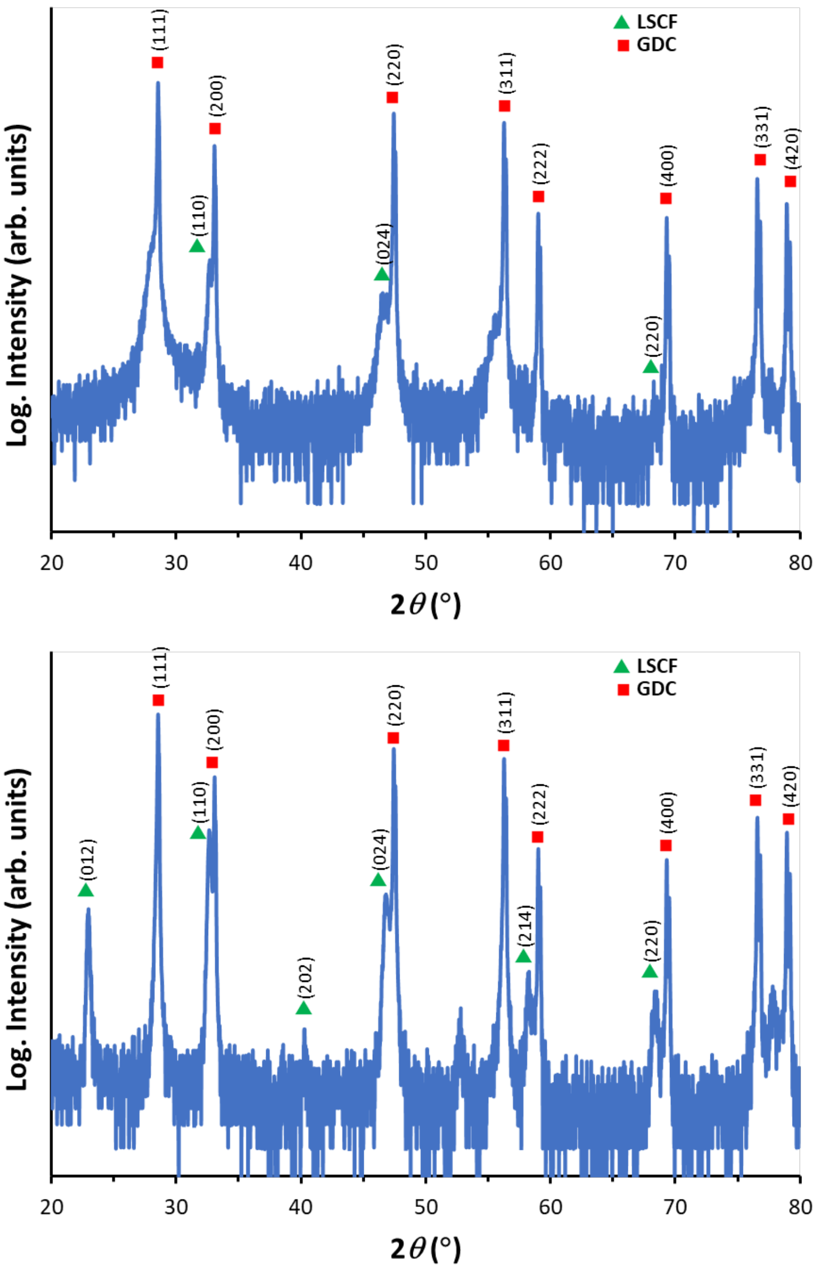

Supplementary Figure 1 Structural characterization using XRD. 2θ-θ XRD patterns obtained for the LSCF-GDC nanocomposite (top) and a reference LSCF film (bottom) deposited using the same PLD conditions. Peaks are indexed to LSCF (▲) (ICSD 186173) and GDC (■) phases (JCPDS 075-0161).

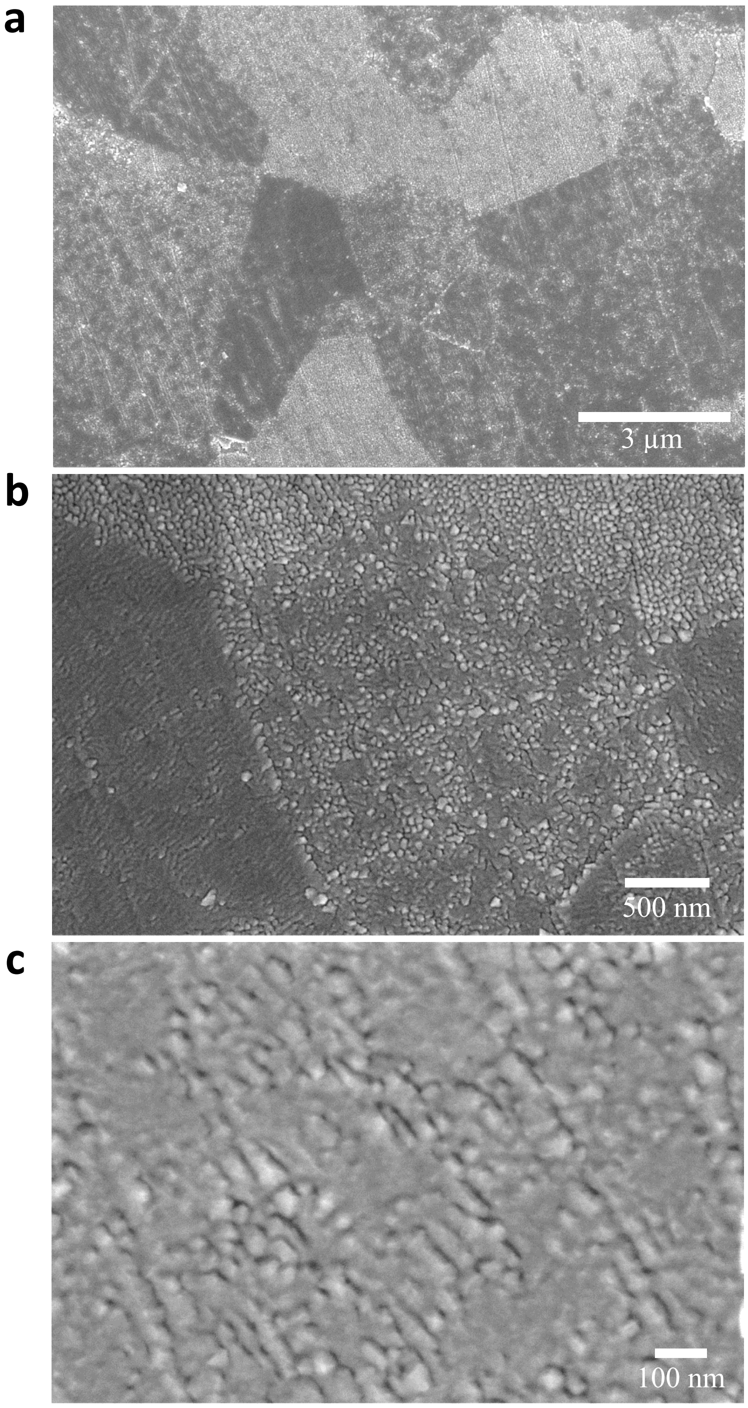

Supplementary Figure 2 Microstructural evaluation of LSCF-GDC nanocomposite film. Representative plan-view SEM (secondary electron image mode) images of various magnifications (**a** 30,000×, **b** 100,000×, **c** 300,000×) obtained for the LSCF-GDC nanocomposite film prepared on GDC electrolyte.

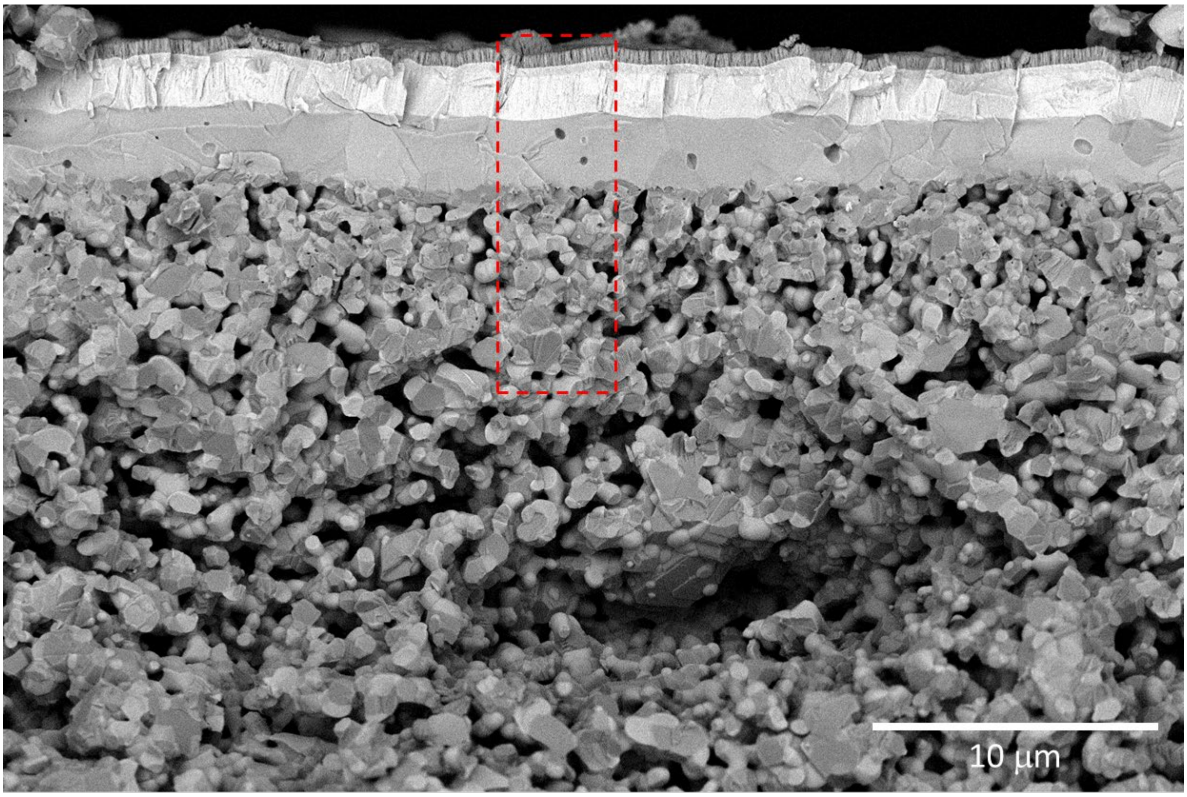

Supplementary Figure 3 Microstructural evaluation of anode-supported cell. Representative low-magnification cross-sectional SEM image (backscattered electron mode) of the anode-supported cell after depositing the PLD layers. The region indicated by a dashed rectangle is shown in higher magnification in Supplementary Fig. 4.

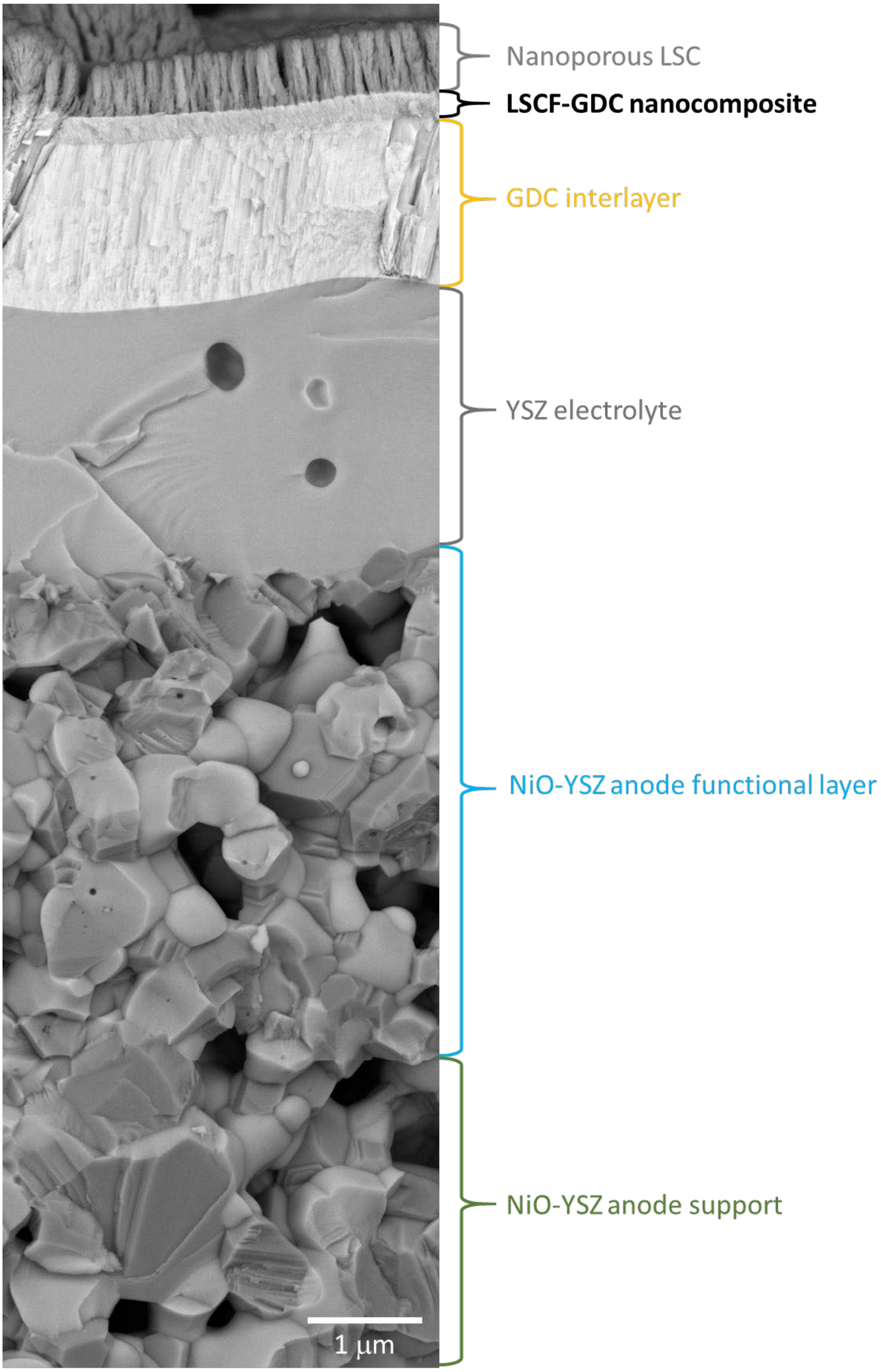

Supplementary Figure 4 Cross-section of anode-supported cell. Composite SEM images showing details of the various layers for the anode-supported cell (region indicated in Supplementary Fig. 3).

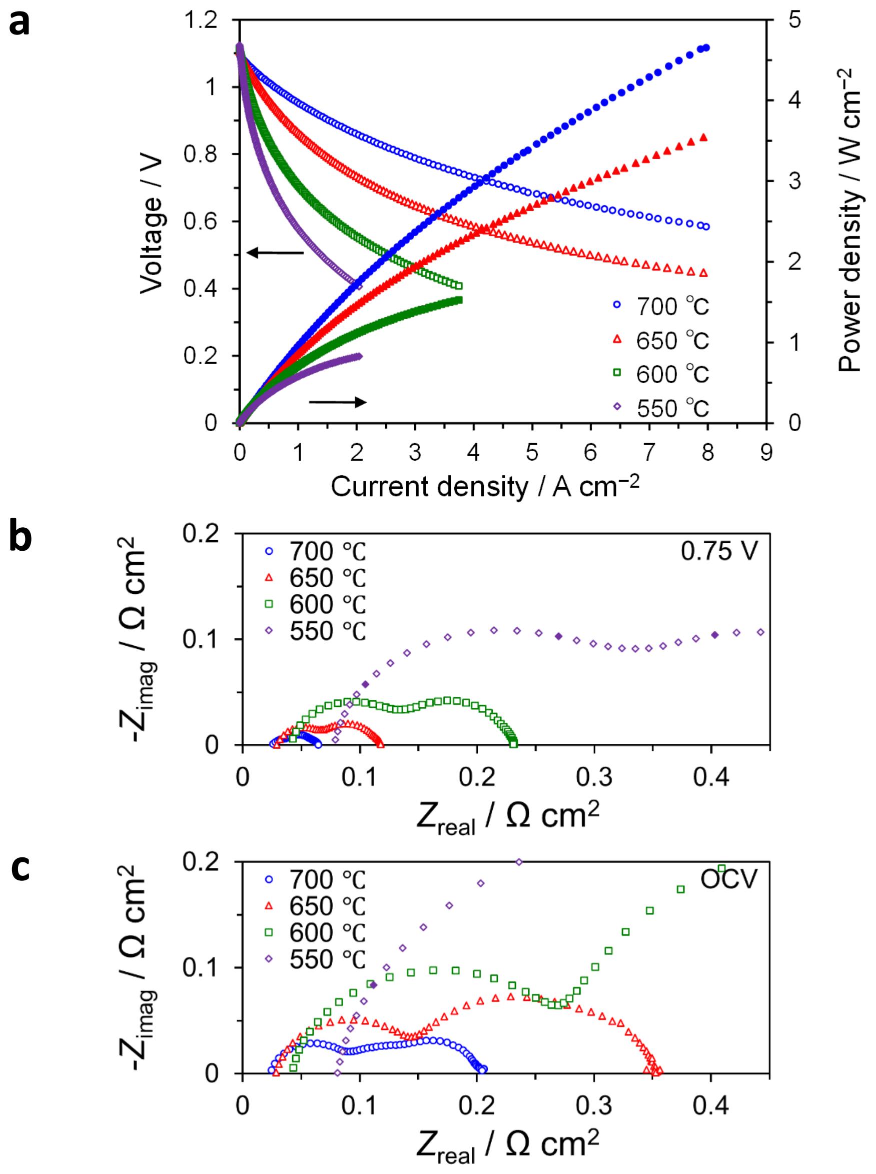

Supplementary Figure 5 Electrochemical performance of anode-supported cell. **a** *I-V* characteristics and power densities evaluated at various temperatures of the anode-supported cell utilizing nanoengineered cathode layer comprised of nanoporous LSC and LSCF-GDC nanocomposite (Sample 9 configuration, see Supplementary Table 1). **b** Impedance spectra for the anode-supported cell at an operating condition of 0.75 V. **c** Impedance spectra for the anode-supported cell at OCV. The results matched very closely those shown in the main text (Fig. 8), indicating excellent reproducibility of the electrochemical performance of the cells. Current collector: Pt paste; active electrode area is 0.283 cm^2.

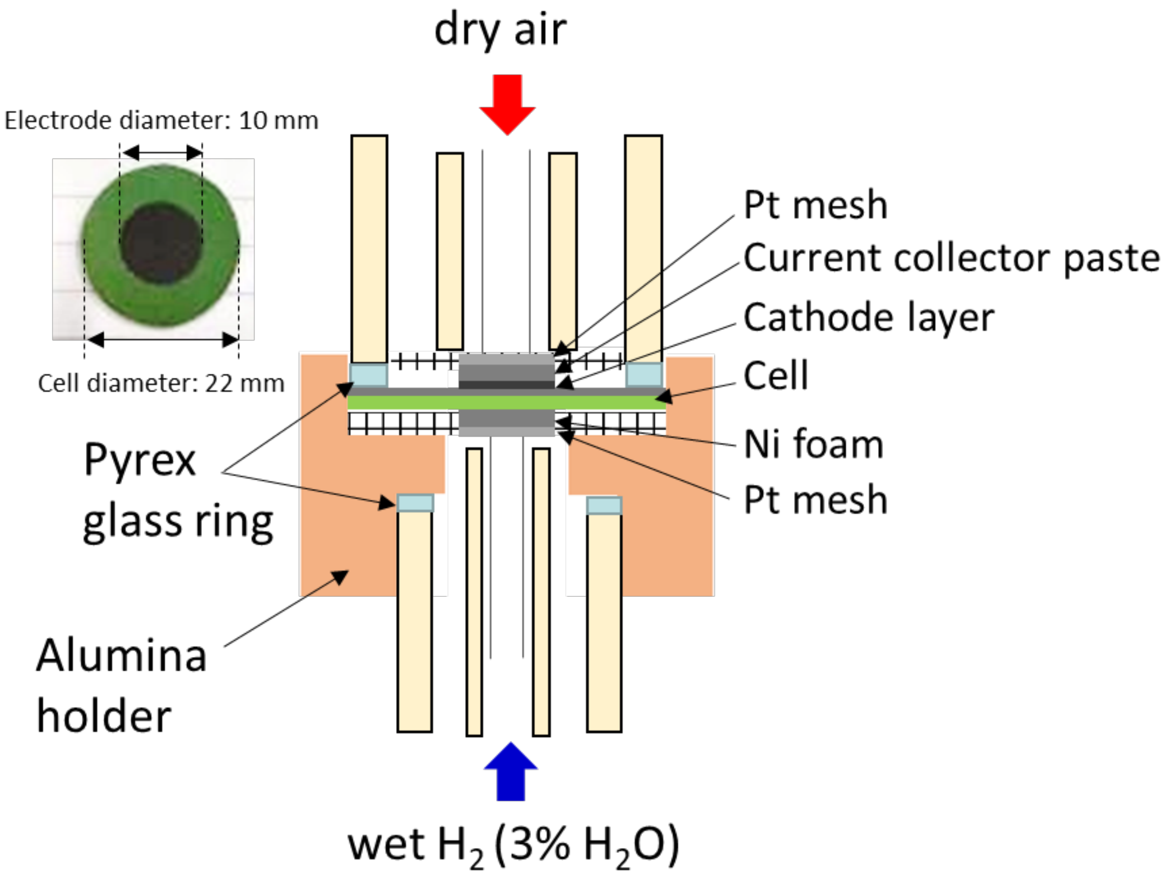

Supplementary Figure 6 Schematic illustration of the electrochemical test set-up. This setup was used for the evaluation of the anode-supported cells (Sample 6, 7, and 8, see Supplementary Table 1). The current collector for the cathode consists of unsintered LSC paste in contact with Pt mesh. Active electrode area is 0.785 cm^2.

Supplementary Table 1. Summary of cell samples in this study.

Sample	Configuration	Cathode	Active area [cm^2]	Electrolyte
1	Symmetrical cell	LSC paste (baseline)	0.785	GDC
2	Symmetrical cell	LSC (~1 μm)*	0.785	GDC
3	Symmetrical cell	LSCF-GDC	0.785	GDC
4	Symmetrical cell	LSCF-GDC + LSC	0.785	GDC
5	Symmetrical cell	LSCF + LSC	0.785	GDC
6	Anode-supported cell	LSCF-GDC	0.785	GDC/YSZ
7	Anode-supported cell	LSC	0.785	GDC/YSZ
8	Anode-supported cell	LSCF-GDC + LSC	0.785	GDC/YSZ
9	Anode-supported cell	LSCF-GDC + LSC	0.283	GDC/YSZ

*nominal thickness of as-grown film
